# Pramipexole Reduces *zif-268* mRNA Expression in Brain Structures involved in the Generation of Harmaline-Induced Tremor

**DOI:** 10.1007/s11064-020-03010-5

**Published:** 2020-03-14

**Authors:** Barbara Kosmowska, Krystyna Ossowska, Jadwiga Wardas

**Affiliations:** Department of Neuropsychopharmacology, Maj Institute of Pharmacology, Polish Academy of Sciences, 12 Smętna Street, 31-343 Kraków, Poland

**Keywords:** Pramipexole, Harmaline-induced tremor, Essential tremor, *zif-268* mRNA

## Abstract

Essential tremor is one of the most common neurological disorders, however, it is not sufficiently controlled with currently available pharmacotherapy. Our recent study has shown that pramipexole, a drug efficient in inhibiting parkinsonian tremor, reduced the harmaline-induced tremor in rats, generally accepted to be a model of essential tremor. The aim of the present study was to investigate brain targets for the tremorolytic effect of pramipexole by determination of the early activity-dependent gene *zif-268* mRNA expression. Tremor in rats was induced by harmaline administered at a dose of 15 mg/kg *ip*. Pramipexole was administered at a low dose of 0.1 mg/kg *sc*. Tremor was measured by Force Plate Actimeters where four force transducers located below the corners of the plate tracked the animal’s position on a Cartesian plane. The *zif-268* mRNA expression was analyzed by in situ hybridization in brain slices. Harmaline induced tremor and increased *zif-268* mRNA levels in the inferior olive, cerebellar cortex, ventroanterior/ventrolateral thalamic nuclei and motor cortex. Pramipexole reversed both the harmaline-induced tremor and the increase in *zif-268* mRNA expression in the inferior olive, cerebellar cortex and motor cortex. Moreover, the tremor intensity correlated positively with *zif-268* mRNA expression in the above structures. The present results seem to suggest that the tremorolytic effect of pramipexole is related to the modulation of the harmaline-increased neuronal activity in the tremor network which includes the inferior olive, cerebellar cortex and motor cortex. Potential mechanisms underlying the above pramipexole action are discussed.

## Introduction

Essential tremor (ET) is one of the most common neurological disorders and the most frequently occurring, apart from the restless leg syndrome, movement disorder in adults [1]. Currently, ET treatment is based mainly on pharmacotherapy and the first-line drugs include propranolol and primidone, which have good efficacy, but in more than 50% of patients produce serious side effects, such as hypotension, dizziness, bradycardia, cognitive impairment, fatigue or erectile dysfunction [2]. Therefore, it is still vital to continue to search for new safer and more efficacious drugs for ET. The most commonly used animal model to search for substances with anti-ET potential is based on tremor induction by acute harmaline administration. Harmaline produces kinetic/postural tremor of the whole body with the peak of oscillation frequency between 10–12 Hz in rats [3–8]. The mechanism of harmaline-induced tremor includes abnormal synchronous activation of the olivo-cerebellar glutamatergic climbing fibers [9] and enhancement of the complex spike discharge of the Purkinje cells (PCs) of the cerebellar cortex [10]. The climbing fibers are also connected directly to the deep cerebellar nuclei (DCN) which send glutamatergic projections to the ventral motor thalamic nuclei [11–13], from where the glutamatergic signal continues to be transmitted to the motor cortex [14]. All of the above structures were proven to be involved in the generation and spread of harmaline tremor by an enhanced expression of different neuronal activity markers (*c-fos*, *zif-268*) [6, 15, 16], and in the case of the cerebellum [16, 17] and motor thalamus [18], also by an excessive glutamate release.

However, it should be noted that the glutamatergic system, which directly connects the above structures, is one of the most important but not the only one involved in the modulation of harmaline tremor. There is a strong evidence to suggest that ligands of different dopamine receptors can affect this behavior. Paterson et al. [4] observed that apomorphine (a non-selective dopamine receptors’ agonist) and quinpirole (D2/D3 receptor agonist) decreased harmaline tremor, while SKF 82,958 (D1 receptor agonist) and raclopride (D2 receptor antagonist) had no effect on it. What is more, they showed that GBR 12,909 [dopamine transporter (DAT) inhibitor] diminished the tremor, but only at the middle dose (the lower and higher doses were ineffective). On the other hand, our own results indicate that apomorphine increases tremor induced by harmaline [8], while pramipexole and 7-OH-DPAT, which are preferential D3 receptor agonists, visibly inhibit this behavior at low doses [5].

At present there is still too few data to establish the contribution of dopaminergic transmission to ET. In the DaTscan neuroimaging study in patients with ET, a correct image of the brain is observed, with no changes in the binding to DAT [19, 20]. On the other hand, the results of clinical trials conducted on a small group of patients with ET indicate the potentially important role of dopaminergic transmission in ET modulation. Herceg et al. [21] in an open-label pilot study observed that pramipexole, which is primarily used to control tremor in Parkinson’s disease, administered for 16 weeks, significantly reduced the severity of tremor by 52%, and after completion of the trial 51.7% of the patients enrolled wanted to remain on pramipexole treatment. Therefore, this clinical result taken together with our research in the harmaline model [5], confirm the potential effectiveness of pramipexole in the treatment of ET.

The aim of the present study was to identify the brain structures involved in the mechanisms of the tremorolytic action of pramipexole in the harmaline-induced model of ET in rats. Determination of *zif-268* mRNA expression level is one of the tools used to study neuronal responsiveness in the selected brain structures. The *zif-268* gene (also known as Egr-1, NGFI-A, Krox-24) belongs to the family of immediate early genes (IEGs) encoding various regulatory transcription factors which modulate the expression of late-response target genes involved in many processes, such as cell growth, differentiation, apoptosis and others (for Refs. [22, 23]). It is worth noting that the expression of *zif-268,* under basal conditions, is relatively high in many rat brain structures [22] and has been proven to be associated with normal synaptic activity. However, it can be rapidly enhanced by a variety of stimuli, both physiological (such as neurotransmitters or growth factors) and pathological (like seizures, ischemia or cellular stress) [22, 24, 25]. The level of *zif-268* expression is closely related to factors including physiological excitation of NMDA and non-NMDA glutamate receptors and can also be induced by the blockade of GABA-A receptors, stimulation of dopamine D1 or blockade of dopamine D2 receptors [22]. Brain distribution and the amount of *zif-268* mRNA and protein show close correspondence which may indicate that expression of this gene is regulated principally at the level of transcription (for Refs. [22, 23]). Basal levels of *zif-268* mRNA have been detected in many structures in the rat brain, such as the cerebral cortex, hippocampus, striatum, cerebellar cortex and others [22].

Since *zif-268* expression is generally considered a sensitive neurochemical marker useful in assessing the response of neurons in the examined area of the brain to given stimuli (for Refs. [22, 23]), we decided to examine the influence of pramipexole on the level of its mRNA in brain areas which are proven to be affected by harmaline [6], i.e. in the inferior olive (IO), cerebellar cortex, ventroanterior/ventrolateral motor thalamic nuclei (VA/VL) and motor cortex. To correlate the changes in *zif-268 mRNA* expression with tremor intensity, behavioral experiments with measurement of tremor and locomotor activity parameters were also conducted in the same animals.

## Materials and Methods

### Animals

Adult male rats (Wistar Han, 300–340 g) were obtained from Charles River (Germany) and prior to the experiment housed in the animal facility at a constant temperature and humidity under a regular light/dark cycle (light 7 AM–7 PM) with free access to food and water. Behavioral tests were performed during the light phase between 8 a.m. and 3 p.m. The experiments were carried out in accordance with the European Union legislation (Directive of September 22, 2010, no. 2010/63/EU) and were approved by Local Ethics Committee at the Institute of Pharmacology, Polish Academy of Sciences (permissions no: 1069/2013, 1290/2016). All efforts were made to minimize the number and suffering of animals used.

### Drugs

Harmaline hydrochloride dihydrate (Sigma-Aldrich, USA) and pramipexole dihydrochloride (Abcam, UK) were dissolved in sterile redistilled water for injection (Polpharma, Poland). Animals were randomly assigned to four experimental groups: SOLV (water *sc* + water *ip*), HARM (water *sc* + harmaline 15 mg/kg *ip*), PRA (pramipexole 0.1 mg/kg *sc* + water *ip*) and PRA + HARM (pramipexole 0.1 mg/kg *sc* + harmaline 15 mg/kg *ip*). Pramipexole or redistilled water was administered 30 min before harmaline. The doses and timeline of administration of compounds were based on our previous studies [5, 6, 8]. The behavioral tests using force plate actimeters (FPA) started immediately after harmaline injection and lasted 60 min. After the tremor measurement, animals were decapitated and the whole brains were isolated for in situ hybridization procedure.

### In Situ Hybridization

Quantitative in situ hybridization of *zif-268* mRNA in rat brain structures was performed according to Kosmowska et al. [6]. The 45-mer synthetic oligonucleotide probe used was complementary to the 3–47 bp of the rat *zif-268* mRNA gene (NM_012551.2, gi: 148747152). Sequence homology with other genes was verified using a GenBank BLAST program. The probe was labeled with [^35^S]dATP (1000 Ci/mmol, Hartmann Analytics, GmbH, Germany) using terminal deoxynucleotidyl transferase enzyme (Thermo Fisher Scientific Inc., USA) to obtain a specific activity of about 5–6 × 10^5^ cpm/μl, and then purified using a phenol:chloroform standard protocol.

The brain tissue sections were incubated in a hybridization buffer with the radiolabeled oligonucleotide (5 × 10^5^ cpm per tissue section) for 20 h at 37 °C in humidified chambers, washed (2 × SSC at 42 °C and 1 × SSC at a room temperature), dehydrated, air-dried, and exposed to a Kodak BioMax MR film (Sigma Aldrich, USA) for 4 weeks at 4 °C.

Signal density [the mean optical density minus background (Q-BG) per unit area (pixel^2^)] was measured in the scanned images using Multi Gauge 3.0 program (Fujifilm Europe, GmbH, Poland). The mRNA expression was estimated in the motor cortex (M1/M2) at four consecutive levels [level 1: A = 1.68 to 0.96 mm; level 2: A =  − 0.72 to − 1.20 mm; level 3: A =  − 1.56 to − 2.28 mm; level 4: A =  − 2.64 to − 3.60 mm from the bregma], VA/VL [A =  − 1.56 to − 2.28 mm from the bregma], cerebellum (lobules 1–10) [A =  − 10.08 to − 13.68 mm from the bregma], and in IO [A =  − 13.08 to − 13.68 mm from the bregma], according to Paxinos and Watson [26].

### Force Plate Actimeters (FPA)

Measurement of tremor and locomotor activity was performed according to Kosmowska et al. [5, 6] and Ossowska et al. [8]. FPA apparatus consists of a measuring cage placed in a sound-attenuating chamber. Four force transducers placed under the corners of the measuring cage‘s floor allow for recording the animal position on a Cartesian plane, tracking its movement across the floor and measuring the force exerted on the plate. Data were collected during time units of 10.24 s (“frames”) with the sampling frequency of 100 points/second. Tremor was analyzed using Fast Fourier Transform on each frame of the experiment. The resulting power spectra were subjected to logarithmic transformation (log10) and averaged over two consecutive 180-frame series (30.72 min) to give the following parameters: AP1—averaged power in the frequency band I (0–8 Hz), AP2—averaged power in the frequency band II (9–15 Hz). The total distance traveled during two consecutive 180-frame series in millimeters was used as a measure of locomotor activity. Because the harmaline tremor is intensified during movements, to analyze the relationship between tremor and motility of rats, the distance was further divided by 10,000 and the AP2/distance ratio was calculated for the animal group with co-administration of pramipexole and harmaline vs*.* rats injected only with harmaline.

### Statistics

Statistical analyses were carried out using the Statistica v.10 software (StatSoft Inc., USA). In the behavioral experiments, ANOVA for repeated measures was used, while in situ hybridization data were analyzed by factorial ANOVA. For individual comparisons between groups, the LSD post-hoc test was used. To evaluate the relationship between the activation of *zif-268* mRNA and the intensity of tremor in the same animals, the Pearson’s linear correlation coefficient was calculated for hybridization signal density and AP2 parameter in different brain structures.

## Results

### The Inhibitory Effect of Pramipexole on the Harmaline-Induced *zif-268* mRNA Expression

**Fig. 1:**
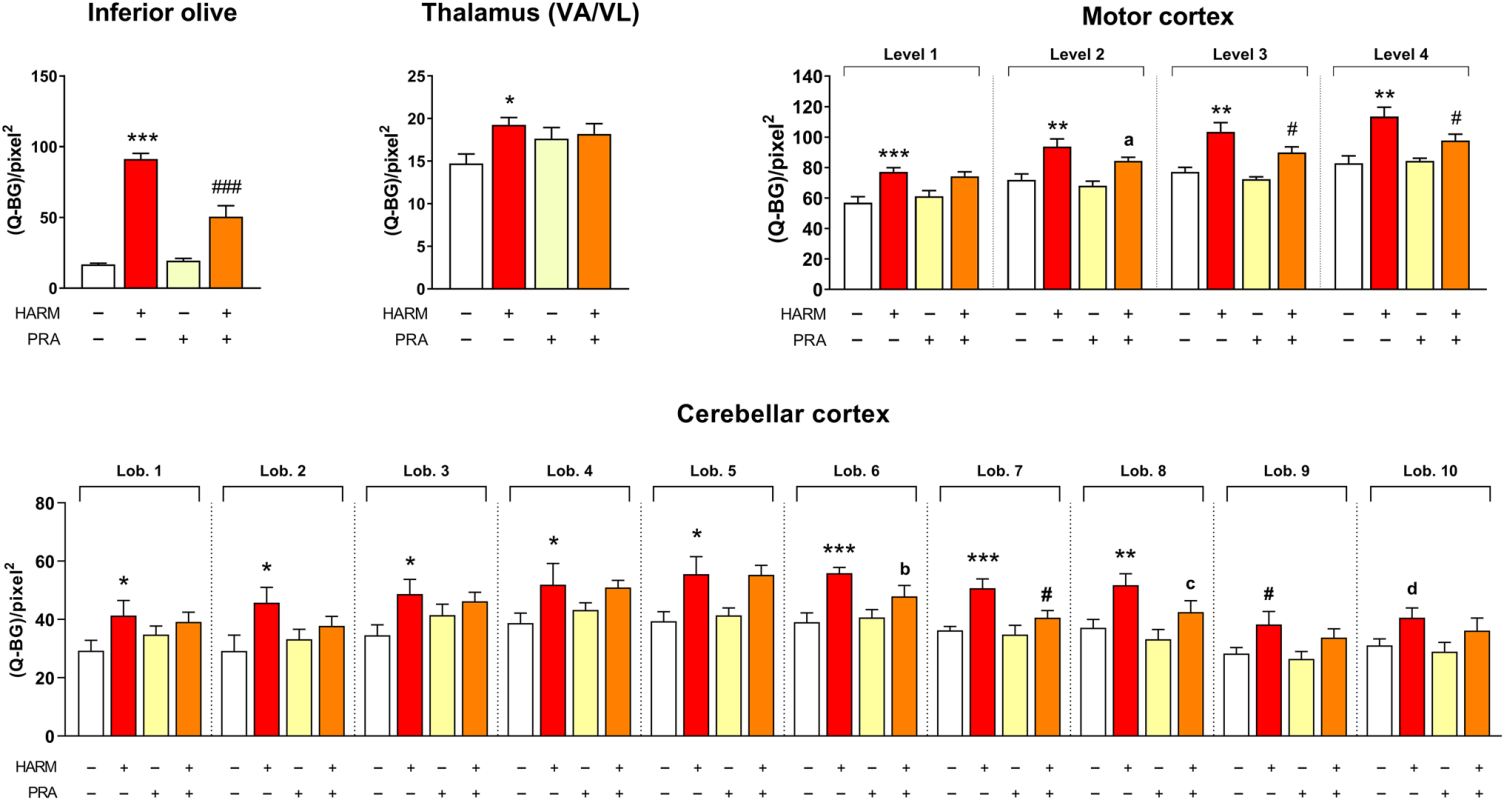
Reversal of the harmaline-increased *zif-268* mRNA expression by pramipexole. *HARM* harmaline 15 mg/kg, *PRA* pramipexole 0.1 mg/kg, *SOLV* solvent, redistilled water; *(Q-BG)/pixel2* the mean optical density minus background (Q-BG) per unit area (pixel^2^), *lob. 1–10* cerebellar lobules 1–10, *VA/VL* ventroanterior/ventrolateral thalamic nuclei, according to Paxinos and Watson [26]. The number of animals: SOLV, n = 7–8; HARM, n = 8; PRA, n = 7–8; PRA + HARM, n = 5–8. Factorial ANOVA with regard to the inferior olive (HARM effect: F[1,54] = 126.111, p = 0.001; PRA effect: F[1,54] = 16.335, p = 0.001), thalamus (HARM effect: F[1,54] = 4.255, p = 0.043), motor cortex (HARM effect: F[1,43]–F[1,51] = 23.398–31,517, p = 0.001; PRA effect for level 3: F[1,51] = 5529, p = 0.022); cerebellar cortex (HARM effect: F[1,27] = 4.350–15.475, p = 0.001–0.047; PRA effect for lobule 7: F[1,27] = 4.426, p = 0.045, for lobule 8: F[1,27] = 3.268, p = 0.082). LSD post-hoc test: *p ≤ 0.05, **p ≤ 0.01, ***p ≤ 0.001, ^d^p = 0.068 vs*.* SOLV; ^#^p ≤ 0.05, ^a^p = 0.064, ^b^p = 0.067, ^c^p = 0.099 vs. HARM

Harmaline significantly increased the *zif-268* mRNA expression in all analyzed brain structures: IO, cerebellar cortex (lobules 1–9; tendency in lobule 10, p = 0.068), VA/VL and motor cortex in comparison to control group (Fig. 1). The overall effect of harmaline was, therefore, similar as in our previous research [6].

Pramipexole given alone did not influence the *zif-268* mRNA expression in any of the above structures in naive rats, but when administered 30 min before harmaline, it inhibited the harmaline-induced effect by decreasing the *zif-268* mRNA expression in IO, motor cortex (level 3 and 4; tendency in level 2, p = 0.064) and selected lobules of cerebellar cortex (lobules 7, tendency in lobule 6 with p = 0.067 and 8 with p = 0.099) but did not affect the gene expression in VA/VL (Fig. 1).

### Pramipexole Inhibits Harmaline-Induced Tremor and Locomotor Activity

**Fig. 2:**
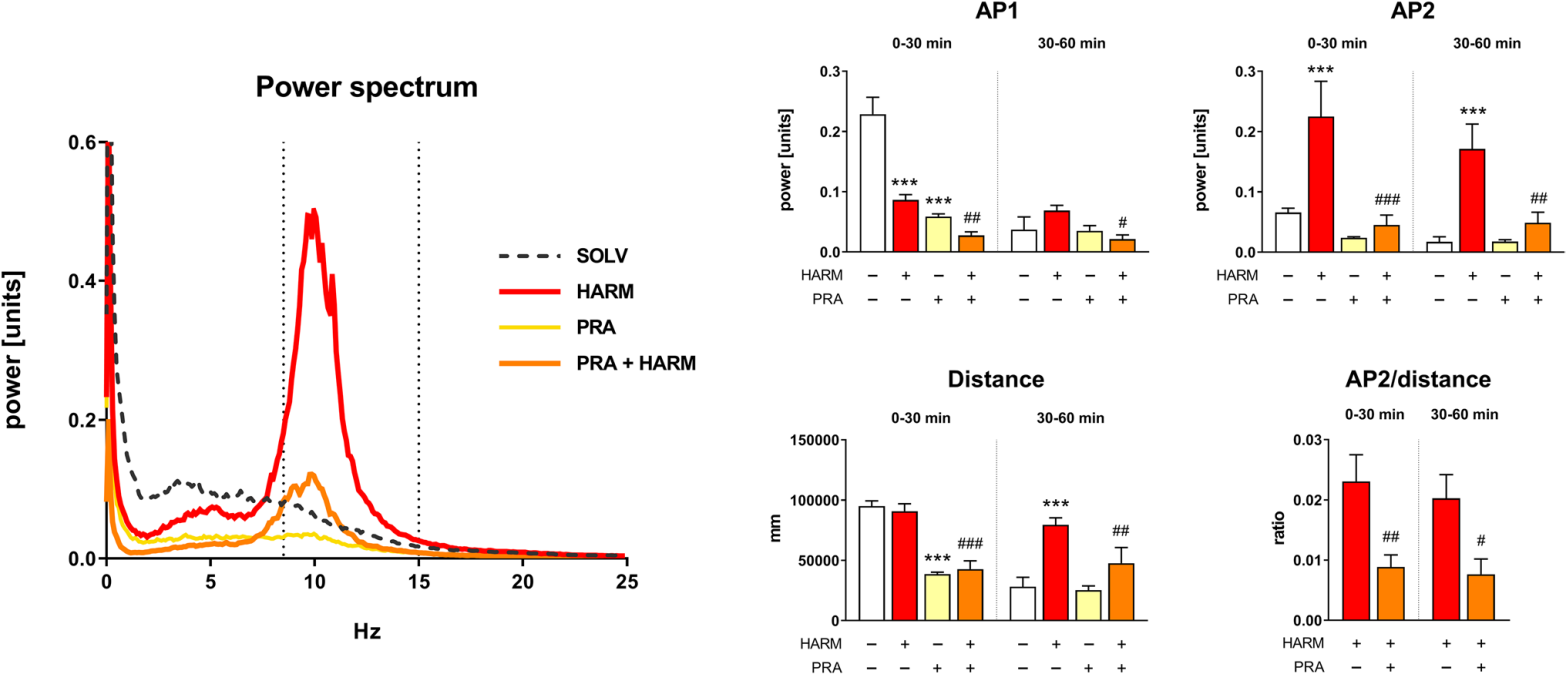
The effect of pramipexole (0.1 mg/kg) on the power spectrum, tremor parameters (AP1, AP2) and locomotor activity (distance) of rats. The power spectrum within a range of 0–25 Hz averaged over the whole measurement period (0–60 min) for all animals is shown. *AP1* power in the 0–8 Hz band, *AP2* power in the 9–15 Hz band. The data are shown as the means ± SEM. The number of animals: SOLV, n = 7; HARM, n = 8; PRA, n = 8; PRA + HARM, n = 8. ANOVA for repeated measures with regard to AP1 (HARM effect: F[1,27] = 13.558, p = 0.001; PRA effect: F[1,27] = 43.279, p = 0.001; time effect: F[1,27] = 63.584, p = 0.001), AP2 (HARM effect: F[1,27] = 12.186, p = 0.002; PRA effect: F[1,27] = 10.778, p = 0.003; time effect: F[1,27] = 7.533, p = 0.011), distance (HARM effect: F[1,27] = 9.181, p = 0.005; PRA effect: F[1,27] = 32.766, p = 0.001; time effect: F[1,27] = 39.143, p = 0.001) and AP2/distance ratio (treatment effect: F[1,14] = 8.254, p = 0.012). LSD post-hoc test: ***p ≤ 0.001 vs. SOLV; ^#^p ≤ 0.05, ^##^p ≤ 0.01, ^###^*p* ≤ 0.001 vs. HARM. For further details, see Fig. 1

Harmaline (15 mg/kg), like in all our previous studies [5, 6, 8], induced generalized tremor which appeared already a few minutes after administration and was manifested by a quick increase in power within frequency band of 9–15 Hz (AP2), with the peak for frequencies around 10 Hz (Fig. 2), which persisted until the end of measurement, i.e. for the entire 60 min. Harmaline decreased also the power within 0–8 Hz frequency band (AP1), but this effect was observed only within the first 30 min (Fig. 2). With regard to locomotor activity, harmaline had no effect on distance traveled within the first 30 min of measurement, but enhanced it between 30 and 60 min in comparison to control animals (Fig. 2).

In harmaline-treated rats, pramipexole showed a significant tremorolytic action by lowering AP2 (0–60 min) by approximately 76%. Additionally, pramipexole administered before harmaline decreased also AP1 and distance parameters (0–60 min) (Fig. 2).

Pramipexole given alone to naive rats only initially (0–30 min) decreased AP1 and distance parameters, but had no effect on AP2 (Fig. 2).

### The Correlation Between the *zif-268* mRNA Expression and the Intensity of Tremor

**Fig. 3:**
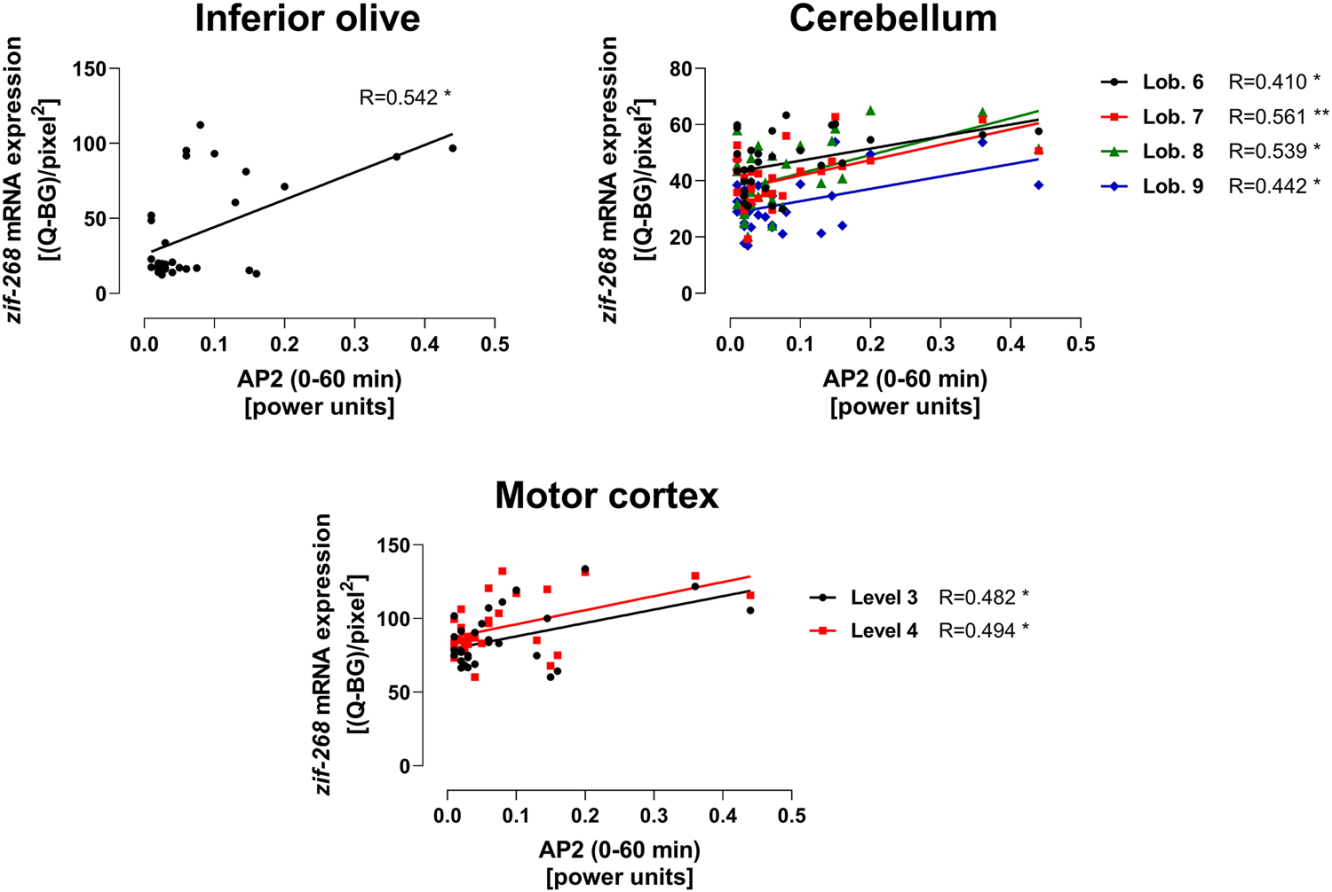
The correlation between the zif-268 mRNA expression in different brain structures and the tremor intensity. The total number of animals used for correlation analysis: N = 27–31 (SOLV, n = 7–8; HARM, n = 7–8; PRA, n = 7–8; PRA + HARM, n = 5–8). Pearson’s correlation coefficient for AP2 and *zif-268* mRNA expression in: inferior olive, R = 0.542; lob. 6–9 of cerebellar cortex, R = 0.410–0.561; level 3 and 4 of motor cortex, R = 0.482–0.494 (*p ≤ 0.05, **p ≤ 0.01). For further details, see Fig. 1

The positive correlation between the *zif-268* mRNA activation (hybridization signal density) and the tremor intensity (AP2 parameter) was observed in the IO, lobules 6–9 of the cerebellar cortex and at level 3 and 4 of the motor cortex, but not in the VA/VL (Fig. 3).

## Discussion

Peripheral harmaline administration induces a rapid increase in the expression level of early response genes, such as *c-fos* or *zif-268*, in the IO and cerebellar cortex [6, 15, 16, 27] as well as in the VA/VL thalamic nuclei and motor cortex [6, 18], indicating the involvement of olivo-cerebello-thalamo-cortical pathway in the generation and spread of tremor induced by this compound. Therefore, in the present study, to identify the brain areas involved in the tremorolytic action of pramipexole, we analyzed its effect on harmaline-induced elevation of *zif-268* mRNA expression in the structures mentioned above (IO, cerebellar cortex, VA/VL thalamic nuclei and motor cortex). Our results indicated that harmaline enhanced the *zif-268* mRNA expression in all the structures involved in the tremor network and that this effect was reversed by pramipexole in all these regions, except for VA/VL.

In order to correlate the *zif-268* mRNA expression data with tremor intensity, we measured also the effect of pramipexole on harmaline-induced tremor parameters using FPA apparatus, in the same animals. The present behavioral experiments confirmed our earlier results that pramipexole given in the low dose (0.1 mg/kg) strongly reduced the tremor induced by harmaline by reversing the harmaline-induced increase in power within 9–15 Hz frequency band (AP2) [5]. Analysis of Pearson’s linear correlation coefficient revealed that in the brain structures where the effect of both harmaline and pramipexole on *zif-268* mRNA expression was demonstrated, there was an evident positive correlation between the *zif-268* mRNA activation and the intensity of tremor (AP2 parameter). This result clearly indicates importance of the IO, cerebellar cortex and motor cortex for tremorolytic effect of pramipexole, however, specific mechanisms underlying the relationship between the above neurochemical marker and tremor remain to be determined.

In addition to tremor inhibition, the above dose of pramipexole reduced also exploratory locomotor activity measured as a distance traveled by harmaline-treated rats, lowering both distance and AP1 (power within 0–8 Hz frequency band) parameters. Since the harmaline-induced tremor has a kinetic character, one might suppose that its reduction is the result of diminished motility. It seems that such hypothesis can be refuted because the analysis of AP2/distance ratio showed a statistically significant decrease in this ratio in the pramipexole + harmaline-treated group in comparison to that treated with harmaline alone. This result indicates that pramipexole had a stronger effect on tremor (measured by AP2) than on locomotor activity (distance), therefore, these two actions appear to be independent. Moreover, a similar effect (decreased AP2/distance ratio) was already observed by Ossowska et al. [8] after propranolol, a well-known drug efficient in treating ET in humans.

The mechanisms underlying the tremorolytic properties of pramipexole in the harmaline model are unclear at present. Pramipexole binds preferentially to dopamine D3 receptors in low nanomolar concentrations (Ki/Kd = 0.2–10 nM), that are 6–95 times lower than those necessary for occupancy of dopamine D2 receptors [28–31]. Involvement of dopamine D3 receptors in the tremorolytic properties of this drug was initially supposed because of a similar effect of 7-OH-DPAT, another D3 receptor-preferring agonist, observed in our previous study [5]. Moreover, pramipexole in the dose of 0.1 mg/kg occupied D3 receptors in the cerebellar lobules 9–10 [32], where they are abundant, especially in the Purkinje cells [33, 34], but not D2 dopamine receptors in vivo [32]. However, the present results show that the inhibitory effect of pramipexole on *zif-268* mRNA expression in harmaline-treated rats was not observed in lobules 9 and 10 but in lobules 6–8, where the levels of dopamine D3 receptors are much lower [34, 35]. Moreover, our previous study showed that the tremorolytic effect of pramipexole was not inhibited by the selective antagonists of D3 receptors (SB-277011-A, SR-21502), amisulpride (an antagonist of D2 and D3 autoreceptors), and haloperidol (a D2-like antagonist) administered at a postsynaptic dose [5]. Importantly, a decrease in locomotor activity and operant responding to a conditioned reinforcer induced by 0.1 mg/kg of pramipexole in rats was not reversed by SB-277011-A and such inhibitory effect was present in D3 knockout mice [32]. All these data challenged the role of dopamine D3 (and D2) receptors in various behavioral effects of the dose of 0.1 mg/kg of pramipexole.

Binding studies have indicated that besides dopamine D3 and D2 receptors, pramipexole binds to dopamine D4 receptors with nanomolar affinity [29, 31]. Since pharmacokinetic studies have indicated that this drug administered in low doses achieves nanomolar concentrations in extracellular fluid of the rat brain [36], the contribution of D4 receptors to its behavioral effects is likely. In fact, our preliminary results (Kosmowska et al. unpublished) have shown that a decrease in locomotor activity induced by pramipexole administered at the dose of 0.1 mg/kg in harmaline-treated animals was reversed by the selective D4 receptor antagonist L745–870. Moreover, a recent study has demonstrated that striatal perfusion of pramipexole reduced optogenetically-induced glutamate release from the corticostriatal terminals in vivo which was counteracted also by L745–870 [37]. The latter result suggested a contribution of presynaptic D4 receptors localized on corticostriatal terminals [38] in the above effect of pramipexole [37]. Since glutamate is an important factor activating *zif-268* expression in the brain (for Refs. [22, 23, 25]), a potential inhibitory action of pramipexole on glutamate release in the brain structures involved in the harmaline-induced tremor (IO, cerebellar cortex and motor cortex) might explain its decreasing effects on this early gene, as well as tremor, observed in the present study and previously [5]. Anatomical localization of dopamine D4 receptors might support this assumption. These receptors are abundant in pyramidal and non-pyramidal neurons of the cerebral cortex, especially in its frontal regions, which provide glutamatergic projections not only to the striatum [38, 39] but to the IO, as well [40, 41]. Therefore, the presence of D4 receptors on terminals of the cortico-olivary projection is possible. Moreover, these receptors have been suggested to be localized presynaptically on climbing fibers in the cerebellar cortex [35, 39], which, as mentioned before, are crucial for generation of the harmaline tremor.

Besides dopamine receptors belonging to the D2-like family, pramipexole binds to α1, α2A,B and 5-HT1A,B,D receptors, but only in high nanomolar or micromolar concentrations [29, 31, 42]. Therefore, contribution of these receptors to behavioral effects of low doses of this drug seems unlikely.

However, some studies have shown that, besides its dopaminergic agonistic properties, pramipexole influences mitochondrial functions, e.g. it inhibits mitochondrial permeability transition pores, when given in nanomolar concentrations, by binding to the inner side of the mitochondrial membrane [43]. Mitochondrial mechanisms have been suggested to underlie antioxidant, antiapoptotic, neuroprotective efficiency of pramipexole which is visible already after its low doses [44, 45]. It is currently unknown whether such non-dopaminergic mechanism indeed contributes also to the inhibitory influence of pramipexole on harmaline effects described in the present study and earlier [5] and to the tremorolytic effects of this drug in ET [21]. However, since some human studies have indicated that mitochondrial dysfunction could be one of the causative factors of ET [46], a search for mitochondrial targets for tremorolytic drugs may be an interesting option for future research.

## Conclusions

Summing up, our results suggest that the tremorolytic effect of pramipexole is correlated with the reversal of harmaline-enhanced *zif-268* mRNA expression in brain structures involved in tremor network, such as the IO, cerebellar cortex and motor cortex. However, the precise neuronal mechanisms underlying these effects are still unclear and need further investigation.
